# An empirical analysis of the cost of rearing dairy heifers from birth to first calving and the time taken to repay these costs

**DOI:** 10.1017/S1751731117000064

**Published:** 2017-02-08

**Authors:** A. C. Boulton, J. Rushton, D. C. Wathes

**Affiliations:** Department of Production and Population Health, Royal Veterinary College, North Mymms, Hatfield, Hertfordshire AL9 7TA, UK

**Keywords:** heifer, economic analysis, management system, grazing, dairy

## Abstract

Rearing quality dairy heifers is essential to maintain herds by replacing culled cows. Information on the key factors influencing the cost of rearing under different management systems is, however, limited and many farmers are unaware of their true costs. This study determined the cost of rearing heifers from birth to first calving in Great Britain including the cost of mortality, investigated the main factors influencing these costs across differing farming systems and estimated how long it took heifers to repay the cost of rearing on individual farms. Primary data on heifer management from birth to calving was collected through a survey of 101 dairy farms during 2013. Univariate followed by multivariable linear regression was used to analyse the influence of farm factors and key rearing events on costs. An Excel spreadsheet model was developed to determine the time it took for heifers to repay the rearing cost. The mean±SD ages at weaning, conception and calving were 62±13, 509±60 and 784±60 days. The mean total cost of rearing was £1819±387/heifer with a mean daily cost of £2.31±0.41. This included the opportunity cost of the heifer and the mean cost of mortality, which ranged from £103.49 to £146.19/surviving heifer. The multivariable model predicted an increase in mean cost of rearing of £2.87 for each extra day of age at first calving and a decrease in mean cost of £6.06 for each percentile increase in time spent at grass. The model also predicted a decrease in the mean cost of rearing in autumn and spring calving herds of £273.20 and £288.56, respectively, compared with that in all-year-round calving herds. Farms with herd sizes⩾100 had lower mean costs of between £301.75 and £407.83 compared with farms with <100 milking cows. The mean gross margin per heifer was £441.66±304.56 (range £367.63 to £1120.08), with 11 farms experiencing negative gross margins. Most farms repaid the cost of heifer rearing in the first two lactations (range 1 to 6 lactations) with a mean time from first calving until breaking even of 530±293 days. The results of the economic analysis suggest that management decisions on key reproduction events and grazing policy significantly influence the cost of rearing and the time it takes for heifers to start making a profit for the farm.

## Implications

Rearing dairy heifers from birth to first calving is an expensive investment which takes a significant time to repay. Using data from 101 dairy farms, we calculated an average cost for rearing a heifer to calving of £1819, or £2.31/day. It took farms an estimated 530 days, or 1.5 lactations, to repay this cost. Age at first calving (AFC) ranged from 21 to 32 months between farms, with later calving significantly more expensive. Calving at a recommended 23 to 25 months is financially beneficial to the farmer in terms of both initial outlay and number of lactations needed to become profitable.

## Introduction

The global dairy farming industry is under pressure from variable feed costs and farmgate milk prices, leading to poor returns. Within this outlook the replacement stock can often be a secondary consideration, with the limited staff time available being concentrated on management of the milking herd which generate immediate income. Rearing replacement heifers involves a large financial investment, contributing ~20% to the overall expenditure on a dairy operation (Gabler *et al*., 2000). There are significant economic gains from rearing heifers efficiently yet most farms do not calculate the true costs as it is hard to separate the inputs from other aspects of the farm business (Mohd Nor *et al*., 2015). Key elements may be omitted, whereas others such as those of feed, labour or disease may be underestimated (Mohd Nor *et al*., 2012). The rearing period is non-productive financially and its length has a direct effect on both the total cost of rearing and the time taken for the individual heifer to pay this back. A heifer calving at 24 months clearly begins to generate income sooner than one calving at 30 months, although this may require a higher initial outlay on feed costs to ensure an adequate growth rate (Tozer, 2000). The farmer only begins to recover the expense of rearing replacements when the amount of revenue realized from milk sales covers both the fixed and variable costs accrued during the rearing period, and only if revenue from milk is greater than variable costs.

A previous UK study concluded that 15% of liveborn heifers never reached first lactation and 19% only calved once (Brickell *et al*., 2009; Brickell and Wathes, 2011). Any heifer which dies or is culled before calving herself incurs a financial outlay which is not recouped and must be covered by income achieved elsewhere. The recommended AFC from both a biological and financial perspective is 23 to 25 months (Mourits *et al*., 1999; Tozer and Heinrichs, 2001; Ettema and Santos, 2004). This does, however, require an adequate growth rate up to this point (Wathes *et al*., 2014). Heifers calving later have reduced odds of completing first lactation (Bach, 2011; Brickell and Wathes, 2011) and those that go on to further lactations have relatively poor fertility with longer calving intervals (Wathes *et al*., 2014). Producers who continue to rear a heifer with a poor growth rate or repeated incidences of disease are wasting resources as the animal is less likely to calve at an optimal age or to survive beyond a first lactation in order to recover the costs of rearing (Waltner-Toews *et al*., 1986; Bach, 2011; Wathes *et al*., 2014).

The cost of rearing a replacement heifer has been estimated in several previous studies. Using a cost analysis spreadsheet, Gabler *et al*. (2000) determined an average total cost in the United States of $1124.06, ranging from $896.86 to $1305.03. A subsequent study by the same group modelled the impact of various factors on the costs, showing a major reduction as AFC reduced from 29 to 21 months (Tozer and Heinrichs, 2001). Using data from 44 dairy operations in Pennsylvania, Heinrichs *et al*. (2013) estimated a cost of $1803±339 from birth until calving. In the Netherlands, Mohd Nor *et al*. (2012) developed a Monte Carlo simulation model that estimated rearing costs at between €1423 and €1715. This model accounted for probabilities of disease and different growth rates depending on whether the animal was healthy or infected. An economic tool, Jonkos, was developed using worksheets in Microsoft Excel to enable farmers to calculate their own costs (Mohd Nor *et al*., 2015). The average heifer rearing cost for 75 herds in this study was €1790 (range €919 to €3307). In the United Kingdom, the average cost to rear a replacement heifer including its own value has been quoted at £1200 ranging from £1100 to £1500. These figures are, however, from companies based on average costs and assumed farm management practices (Promar International, 2011; Kingshay, 2013).

This empirical study presents an analysis based on detailed costs of rearing dairy heifers as herd replacements in Great Britain acquired through a comprehensive data collection, capture and analysis process. Costs were initially determined over three developmental periods: birth to weaning; weaning to conception and during pregnancy until first calving (Boulton et al., 2015a, 2015b and 2015c). The aims of the present paper were as follows: (1) to determine the true total cost of rearing including the cost of heifer mortality covering the entire period from birth to first calving; (2) to investigate the main factors influencing these costs across differing farming systems; and (3) to estimate how long it took for heifers to repay the cost on individual farms, based on revenue from milk production and the cost of maintenance of the milking cows.

## Material and methods

### Farm selection, questionnaire and data collection

A total of 101 UK dairy farms were visited between March and August 2013. Farmers were recruited through dairy extension services, farm consultancy groups, veterinary practices and social media. The farms were a convenience sample, but farms in England, Scotland and Wales were included in proportion to the number of dairy farms located in each country. An effort was also made to include different types of farm which reflected the demographics of the industry. Good farm records, including access to purchase orders to confirm input costs, were essential for inclusion in the study. An approved questionnaire (Royal Veterinary College Ethics and Welfare Committee number 2013 1199) was completed by a single researcher visiting each farm and conducting an interview. The questionnaire included 124 questions (46 closed, 78 open) and was based on a study of calf and heifer management on Canadian dairy farms (Vasseur *et al*., 2010). Data were collected about on-farm calf and heifer management practices, focussing on the areas of calving, neonatal care, weaning, feeding, housing, health and disease and reproductive management. Actual farm input costs were collected in addition to details of the enterprise farm type (traditional or organic farm), seasonal calving pattern, herd size and predominant breeds kept.

Information from farm records were used to calculate the annual culling and calving rates, calculated as a percentage using the number of cows culled and the number of cows calved in the previous 12 months. The annual stillbirth rate was determined by subtracting the number of liveborn calves from the number of cows calving in the previous 12 months and dividing by the number of cows that calved. Average first lactation and herd yields (L) were taken as the most recently recorded 305-day lactation yields.

### Calculation and analysis of input costs

Three development periods were selected for the initial analysis: birth to weaning, weaning to conception and conception to calving. The length of gestation was fixed at 274 days (9 months), based on the average gestation length found in a study of Holstein cattle (Jousan *et al*., 2005). The amount of time that heifers spent in each period was dependent upon the age at which the heifer was weaned, conceived and then calved. The age of conception was determined by subtracting the gestation length from the AFC. The methods used to calculate the cost of labour, calving, feed, bedding and disinfection of housing, the disposal of still born calves, navel disinfection and dehorning, utilities, building and equipment depreciation, water consumption, health and disease treatment, grazing, movement and transportation, reproductive management, slurry storage and disposal of soiled bedding are described fully in Boulton *et al*. (2015a, 2015b and 2015c).

A cost analysis workbook was developed in Microsoft Excel 2010 to calculate the total cost of rearing from birth to first calving on a per heifer basis on each farm visited. This included the fixed and variable costs of rearing incurred in each period, the interest on capital investment, the opportunity cost of the heifer and the cost of mortality. The opportunity cost was calculated based on a 50% probability of having a heifer calf, the difference in market value of continental beef cross bulls and heifers and purebred dairy bulls and the cost of semen. An average conception rate in dairy cows in the United Kingdom of 35% (Cook, 2009) and a perinatal calf mortality rate of 7.9% (Brickell *et al*. 2009) were also assumed. The mean cost of mortality from birth to calving was calculated using mortality data from a longitudinal study of 19 dairy farms across southern England (Brickell *et al*., 2009; Brickell and Wathes, 2011). To calculate a daily mortality rate, the mortality rate for each of the time periods described in the study was divided by the number of days in each of those periods specific to each farm. The cost of resource used up to the point of death was then apportioned to the remaining cohort of surviving heifers.

A gross margin analysis for the rearing herd was calculated for each farm based on the variable costs of feed, forage, veterinary provision and medicine, reproduction management, bedding and disinfection, water, slurry storage, electricity, transport and registration. The output was calculated as the difference in market value of the animal at the end compared with the beginning of each period, less the cost of mortality. The breakeven point (BEP) for each farm was determined based on the revenue from milk production and the cost of maintenance of the milking cows. The variable cost of maintenance included purchased feed and forage, veterinary services and medicines, bedding, breeding costs and sundries. These were taken from the Agricultural budgeting and costing book (Agro Business Consultants, 2013) from the gross margin calculations for all-year-round calving, autumn calving and spring calving for black and white breeds and the separate gross margins for Jersey cows. Variable costs from the all-year-round herds were applied to the multi-block calving herds as they are the most similar to the input costs in this study. Revenue from milk production was the product of lactation yield (L) and farmgate price (pence per L). The length of lactation was taken to be 305 days followed by a dry period whose length was determined by fertility parameters reported in a study by Cooke *et al*. (2013). Each animal was deemed to have reached its BEP when the income it had generated was sufficient to cover its fixed and variable costs over its lifetime in the herd. For heifers that had not reached their own BEP by the end of the second lactation, fertility measures for second lactation cows were applied to the remaining lactations until BEP was achieved.

### Statistical analysis

Cost data were imported into STATA v12.1 (StataCorp, College Station, TX, USA) for statistical analysis. Normality and heteroskedasticity were examined using the Shapiro–Wilk test and the Breusch–Pagan/Cook–Weisberg, respectively. Explanatory variables were assessed for collinearity, with a score of ⩾0.8 indicating high correlation (Dohoo *et al*., 2009). Variables with *P* values <0.2 in univariable analysis were introduced into the multivariable model. A forward stepwise selection procedure was undertaken to determine the model starting with the variable with the highest *F* statistic and lowest *P* value on univariable analysis. The results of the ANOVA were used to determine the fit of the model. Results are presented as the mean±SD.

## Results

### Herd characteristics

The 101 farms included in this study had an average herd size of 237 (range 10 to 1200). These farms were classified according to breed into six types, Holstein (*n*=40), Holstein-Friesian (*n*=29), Holstein-Friesian cross (*n*=13), crossbred (*n*=4), other pure dairy breed excluding Jersey (*n*=9) and Jersey (*n*=6). The most frequent calving pattern was all-year-round (*n*=73), with 14 autumn calving farms (August to October), 10 multi-block calving farms (each with two distinctive annual calving periods) and five spring calving herds (February to April). The majority of farms were conventional herds with nine farms classified as organic as defined by regulations of the European Union (EC No. 834/2007).

### Rearing costs including heifer mortality

The mean AFC for the farms in the study was 784 days (25.8 months) with a range of 639 to 973 days (21 to 32 months). The duration and costs incurred during each period are summarized in Table 1 with the proportional contribution of input costs summarized in Table 2. Feed was the greatest input cost in all three periods, contributing 36.8±8.2% to the total. This increased to 43.8% overall if grazing was also included. The other main costs were labour (22.3±10.1%), bedding (8.7±4.4%) and disposal of slurry and soiled bedding (7.1±3.8%). The mean cost of heifer mortality (an animal which died before she could calve) was £139.83±10.44/surviving heifer (median £143.30: range £103.49 to £146.19, *n*=101). Mortality had a greater cost implication on smaller herds as the cost was spread over a smaller number of surviving heifers.

**Table 1:** Summary data showing the duration and cost of rearing dairy heifers during each development period taken from a survey of 101 British farms

	Age at end of period (day)		Total cost (£)		Daily cost (£/day)	
Periods	Mean	Range	Mean	Range	Mean	Range
Birth to weaning^1^	62±13	42 to 112	£195.15	£94.64 to 499.80	£3.14	£1.68 to £6.11
Weaning to conception^2^	509±60	365 to 700	£745.94	£295.32 to £1745.85	£1.65	£0.75 to £2.97
Conception to calving^3^	784±60	639 to 973	£450.36	£153.11 to £784.00	£1.64	£0.56 to £2.86

1 Data from Boulton *et al*. (2015a).

2 Data from Boulton *et al*. (2015b).

3 Data from Boulton *et al*. (2015c).

**Table 2:** The mean cost of farm inputs for each development period, calculated as the percentage of the total

	Development period			Total
Input	Birth to weaning (%)	Weaning to conception (%)	Conception to calving (%)	Birth to calving (%)
Feed	46.4	35.6	32.7	36.8
Labour	11.2	24.7	23.8	22.3
Bedding and disinfection	13.6	9.1	6.6	8.7
Slurry and soiled bedding	5.4	4.8	11.2	7.1
Grazing	–	7.0	11.0	6.9
Reproduction	–	8.4	–	4.4
Housing and machinery	4.3	3.2	6.0	4.3
Health and disease	9.6	3.8	3.8	4.1
Water	0.3	2.0	4.2	2.4
Electricity	1.1	2.2	0.8	1.6
Other^1^	8.1	–	–	1.1

1 Other: registration, dehorning, navel disinfection, calving and colostrum management.

The mean cost of rearing a dairy heifer to the point of calving, including fixed and variable costs was £1391.47±362.87 (*n*=101, median £1360.27, range £604.01 to £2482.96). With the addition of capital and opportunity costs, the mean total cost of rearing increased to £1819.01±387.09 (median £1794.55, range £1073.36 to £3070.46). The interest on capital and the opportunity cost together accounted for on average 24.3±5.7% (median 23.4%) of the total cost of rearing.

The mean daily cost of rearing per heifer (including interest and opportunity cost) was £2.31±0.41 (median £2.32: range £1.47 to £3.35). The average cost contributions for each rearing period were 10.8±3.3% from birth to weaning, 40.4±6.0% from weaning to conception and 24.5±4.2% from conception to calving, with median values of 9.9%, 40.7% and 24.5%, respectively. As the birth to weaning period was much shorter, the cost per day at this time was about twice that incurred during the other two periods: birth to weaning, £3.14±0.85/day; weaning to conception, £1.65±0.40/day and conception to calving, £1.64±0.47/day (Table 1).

### Influence of management variables on total cost of rearing

**Figure 1:**
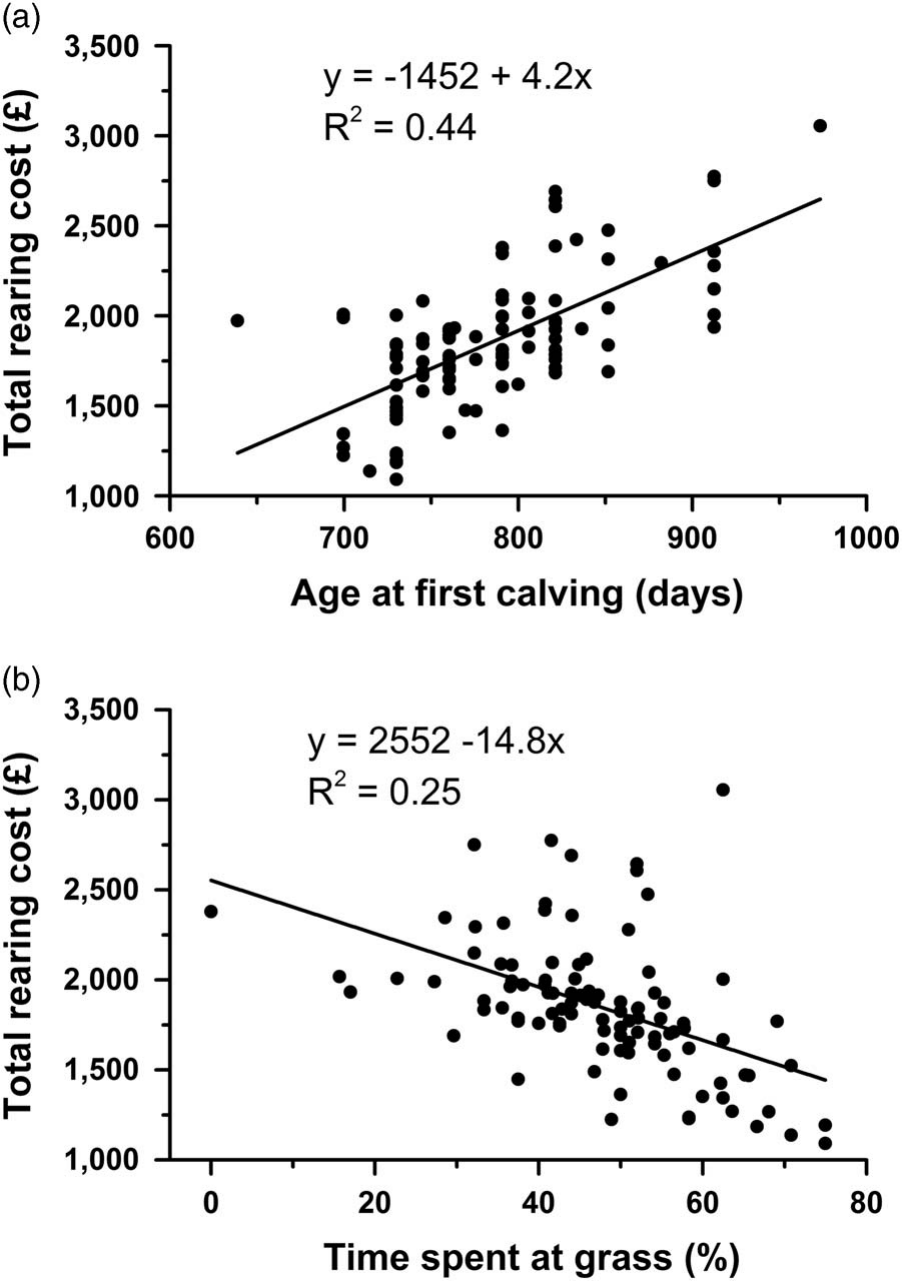
Relationship between the total cost of rearing and (a) age at first calving (days) or (b) the percentage of time that heifers spent at grass. Each symbol represents the data from one farm, *n*=101.

**Figure 2:**
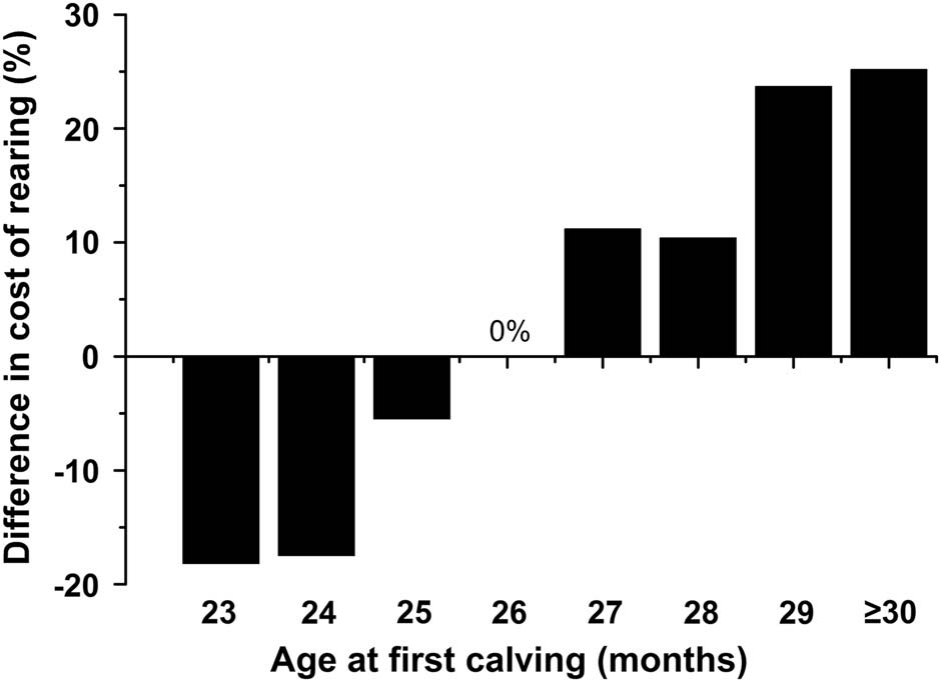
The estimated percentage difference in the total cost of rearing according to age at first calving, with 26 months taken as the base month (0%).

Univariate analysis provided evidence of associations between the total cost of rearing with age at breeding, age at conception, AFC, % time at grass, calving pattern, breed and herd size (Table 3). Age at breeding and age at conception were both major influences on AFC and only the latter is discussed further here. The mean AFC was 25.8 months and AFC accounted for 35% of the variation in the total cost of rearing, with the regression equation indicating that it contributed £3.86/day (Figure 1a). Taking 26 months as the baseline (0%, Figure 2), the mean total cost of rearing decreased by 17.1% for calving at 23 months and increased progressively up to 25.2% for an AFC of ⩾30 months.

**Table 3:** Influence of different exposure variables on the outcome variable of total cost of rearing assessed using one-way ANOVA

Variables	df	*F*	Prob. >*F*	Adjusted *R* ^2^
Age at first calving (day)	1, 99	53.67	<0.0001	0.345
Age at conception (day)	1, 99	50.40	<0.0001	0.331
Age at breeding (day)	1, 99	40.81	<0.0001	0.285
Seasonal calving pattern	3, 97	10.31	<0.0001	0.218
Time at grass (%)	1, 99	23.32	<0.0001	0.182
Breed	5, 95	4.07	0.002	0.133
Region of UK	9, 91	1.61	0.125	0.052
Calving rate (%)	5, 95	1.65	0.155	0.031

**Figure 3:**
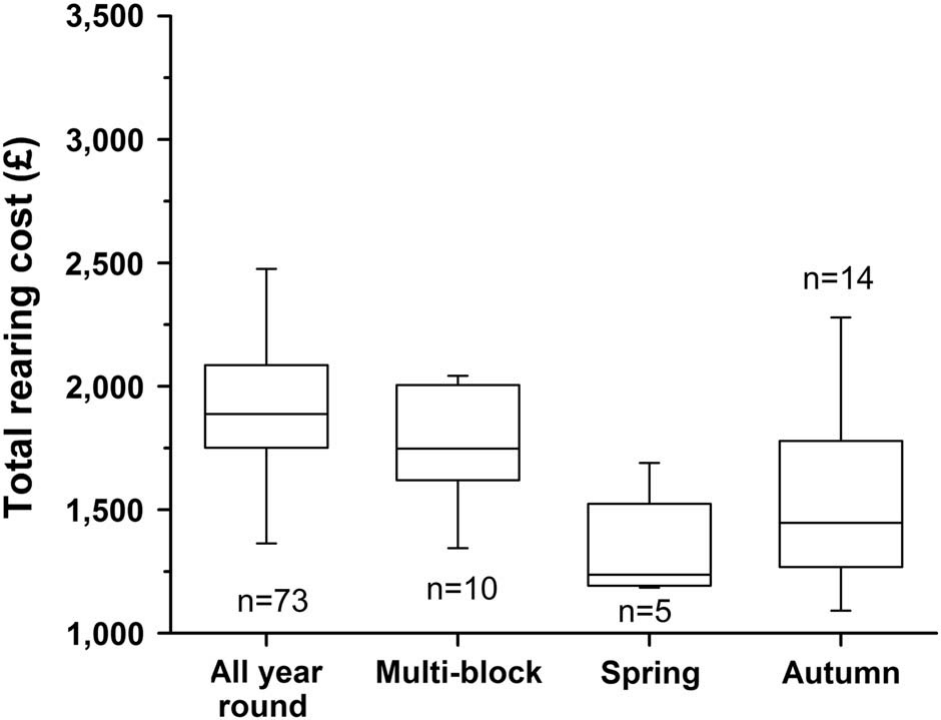
Box and whisker plot of minimum, maximum and median values of total rearing costs by calving pattern. All year round >autumn and spring, *P*<0.0001.

The next most influential variable was calving pattern, showing the importance of seasonality. Spring calving herds had the lowest mean total cost of rearing at £1323.66±161.49, whereas all-year-round calving herds were the highest at £1928.17±360.46. Autumn and multi-block calving herds had intermediate mean total costs at £1495.36±359.09 and £1733.85±198.53, respectively (Figure 3). A regression analysis of calving pattern and total cost of rearing also showed a highly significant difference (*P*<0.0001) between the cost of rearing on all-year-round calving herds and spring and autumn calving herds (data not shown). There was only a weak association between the cost of rearing on all-year-round calving herds and multi-block calving herds (*P*=0.096).

Seasonal effects were also reflected in the time spent at grass, such that for each percentage increase of time the total cost of rearing decreased by £13.29 (Figure 1b, *P*<0.0001). Total rearing costs were also influenced by breed and herd size, and these effects were explored further in the multivariable analysis. A number of other variables investigated did not show a significant relationship to the total cost of rearing using univariate analysis (*P*>0.2). These included enterprise type (traditional or organic), herd status regarding testing for bovine tuberculosis, age at weaning and various measures relating to the performance of the milking herd (herd 305-day yield, first lactation 305-day yield, % culling rate and % stillbirth rate).

### Multivariable analysis

The significant variables from the univariate analysis (Table 3) were carried forward into a multivariable analysis. Age at breeding and age at conception were not included due to their strong collinearity with AFC. The results are presented in Table 4. Age at first calving and % time at grass had the strongest association with total cost of rearing. Allowing for the effect of the other variables, the mean cost of rearing in both spring and autumn calving herds were significantly lower than for all-year-round calving herds. The mean total cost of rearing in multi-block calving herds was, however, not significantly different from all-year-round calving herds in either the univariate or multivariable models.

**Table 4:** Statistical output of multivariable linear regression analysis of total cost of rearing on 101 farms with age at first calving, time spent at grass, calving pattern, breed and herd size^1^

Variables	Coefficient	SEM	*t*	*P*	95% CI	
Age at first calving (day)	2.87	0.53	5.40	<0.0001	1.82	3.93
Time spent grass (%)	−6.06	2.45	−2.47	0.016	−10.93	−1.18
Calving pattern						
Baseline	All year round					
Autumn	−273.20	88.04	−3.10	0.003	−448.22	−98.17
Multi-block	−22.39	95.58	−0.23	0.815	−212.39	167.61
Spring	−288.56	144.74	−1.99	0.049	−576.29	−0.83
Breed						
Baseline	Holstein					
Holstein-Friesian	66.71	66.50	1.00	0.319	−65.49	198.90
Holstein cross	170.39	90.97	1.87	0.064	−10.46	351.24
Friesian cross	−93.82	158.81	−0.59	0.556	−409.51	221.88
Other purebreds	52.78	114.04	0.46	0.645	−173.93	279.48
Jersey	−139.84	121.81	−1.15	0.254	−381.99	102.32
Herd size						
Baseline	⩽99					
100 to 199	−301.75	91.53	−3.30	0.001	−483.70	−119.81
200 to 299	−345.30	99.61	−3.47	0.001	−543.31	−147.28
300 to 399	−407.83	111.65	−3.65	<0.0001	−629.79	−184.87
⩾400	−318.53	146.37	−2.18	0.032	−609.51	−27.54
Constant (baseline)	171.91	459.06	0.37	0.709	−740.67	1084.48

CI=confidence interval.

1 The final model had an *R* ^2^ value of 0.616, *P*<0.0001.

Breed was included in the final model as it was considered to be a possible confounding variable and its overall *F* statistic was significant at *P*=0.002, although not all of the *t*-tests for the levels of the variable were significant. Breed is also important as it affects weight at calving and hence the previous input costs, particularly relating to feed requirements. Including breed in the final model also improved the goodness of fit, increasing the coefficient of determination (*R* ^2^) value from 0.541 to 0.616. Taking pure Holstein as the baseline, costs of rearing were numerically lower in Jersey and Friesian crossbred herds but were somewhat higher in Holstein-Friesian, Holstein cross and other purebred herds. The *t*-tests for each level of herd size were significant in the final model indicating that the mean cost of rearing for each level above 100 cows was significantly different from that of herds with ⩽100 milking cows.

The outcome of the final multivariate model was that AFC, number of milking cows, % time at grass, breed and calving pattern accounted for 61.6% of the variability in total cost of rearing. Adjusting for the effect of the remaining variables in the model, the mean cost of rearing increased by £2.87 for each day increase in AFC, whereas mean rearing costs decreased by £6.06 for each percentile increase in time spent at grass. The predicted decrease in mean cost of rearing on autumn and spring block calving farms compared with all-year-round calving farms was £273.20 and £288.56, respectively. In relation to herd size, the predicted decrease in mean cost of rearing on farms with herd sizes of 100 to 199, 200 to 299, 300 to 399 and ⩾400 cows was £301.75, £345.30, £407.83 and £318.53 compared with a herd size of ⩽99 milking cows.

### Gross margin

The mean gross margin for the entire rearing period was £441.66±304.56 (median £480.33, range £367.63 to +£1120.08, *n*=101). The figures for the three periods separately were as follows: birth to weaning, median £102.19, range £133.89 to+£335.20; weaning to conception, median £335.07, range £485.61 to+£747.16; and conception to calving, median £27.81, range £251.15 to+£260.23. At the end of the period from birth to weaning there were 15 farms with negative gross margins, so the market value of their heifers was lower than their variable input costs. The second period from weaning to conception saw 13 farms with negative gross margins, increasing to 44 for the period from conception to calving. Overall, for the total rearing period, only 11 farms had negative gross margins suggesting that the high market value of freshly calved heifers allowed many farms to make up for high input costs during rearing. In this regard it is important to recognize that a minority of heifers pass through a market system and the prices from the heifer market itself may not accurately represent the market clearing prices for heifers across Great Britain.

### Repayment period

**Figure 4:**
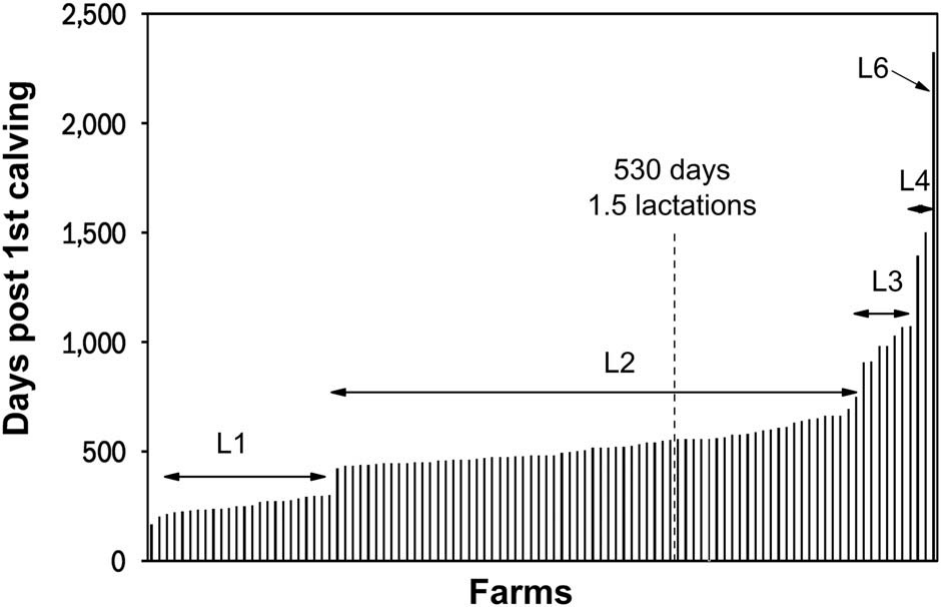
Bar chart showing the number of days after first calving that heifers took to pay back their cost of rearing by lactation number, estimated for each of the 101 farms included in the study. The numbers above the bars (L1 to L6) represent the lactation number at which the breakeven point was reached. The mean time taken over all the farms was 530±293 days.

During the first lactation 23.6% (24/101) of farms were estimated to pay back their cost of rearing. This increased to 91.1% (92/101) by the end of the second lactation, with the remaining 10 farms requiring between three and six lactations to repay their rearing costs (Figure 4). On average heifers paid back their cost of rearing on each farm at 530±293 days after calving for the first time (median 293, range 168 to 2321 days, *n*=101). This translated into ~1.5 lactations before heifers began to make a profit for the farm.

## Discussion

Economic analysis of the rearing period for replacement dairy heifers is important for two main reasons. First, farmers need to appreciate the true total cost and the key components which contribute so that they can appraise their own systems to increase efficiency. Second, it is crucial to understand the long-term consequences of the level of spending on animals of different ages as there is increasing evidence that higher growth rates and reduced disease incidence both improve the survival and productivity of the mature cow within the herd (Waltner-Toews *et al*., 1986; Bach, 2011; Wathes *et al*., 2014). The potentially higher costs of providing better heifer management must, however, be recouped later if the herd as a whole is to remain profitable. This paper has focussed on AFC as a key measure of rearing success, which has a major economic impact. It is, however, important to ensure that the heifers have reached an adequate BW (as a proportion of mature body size) and frame size by the time AFC is reached as this also affects subsequent health and milk production (reviewed by Bach and Ahedo, 2008; Wathes *et al*., 2014).

A number of previous studies outlined in the introduction have estimated the total costs of rearing dairy heifers. Direct comparisons are hard due to differences in global farming systems, currencies and their conversion rates, the year in which the data were obtained and the list of expenses included in the calculations. The results from the present study are generally higher than those previously reported for the United Kingdom. These differences can in part be explained by the inclusion of more headings of expenditure including housing and building maintenance and depreciation, utilities and slurry disposal. This study has taken an economic approach to data collection and analysis rather than a financial cost-based evaluation. Mohd Nor *et al*. (2015) previously reported that 32 out of 37 Dutch dairy herds surveyed underestimated the cost of rearing. The main omissions they found were in the costs of housing and labour, which can be undervalued particularly on family run businesses.

Some clear themes emerge from comparison of the different studies. First, there is general agreement that feed is the major input cost. Feed accounted for 60% and 73% in two studies in the United States (Gabler *et al*., 2000; Heinrichs *et al*., 2013). The proportional rise between 2000 and 2013 was attributed to higher feed purchase costs and the omission of depreciation and manure costs from the later study but both estimates of proportional feed costs were greater than those reported in Europe. The present study and the Dutch study (Mohd Nor *et al*., 2012) both included grazing and the associated costs of land management in their figures and estimated very similar feed costs of 44%. Two other UK studies estimated that feed costs represented 31% and 55% of all costs (Promar International, 2011; Kingshay, 2013). The amount attributed to milk powder by Promar International appeared very low.

Focussing on the British and Dutch studies, where farming systems are likely to be fairly similar, the next main cost was labour, ranging from 32% (Mohd Nor *et al*., 2012), 22.3% (present study) to only 9% (Kingshay, 2013) of all rearing costs. The large range may in part reflect differences in the amount of time during which youngstock were housed. The Dutch study also modelled disease, with the increased labour required to treat sick animals included at 2 and 10 min/calf for animals with pneumonia or scours. A higher incidence of enteric or respiratory disease would therefore translate into a significant extra labour cost. Proportionate housing costs in the Dutch study (Mohd Nor *et al*., 2012) were very similar to those estimated by the present one, with 11.5% and 11.7% of rearing costs, respectively. These were somewhat higher than the costs included under this heading by Promar International (2011, 7.1%) and Kingshay (2013, 8.3%), which both only included bedding. The Dutch study did not specifically state a cost for waste management which may have been included in the barn costs. The cost of health and veterinary treatment was fairly consistent at 4.1% (this study), 3.1% (Mohd Nor *et al*., 2012) and 2.8% (Promar International, 2011).

Expenses associated with reproduction were also in a similar range (present study, 4.4%; Promar International, 2011, 3.8%; Mohd Nor *et al*., 2012, 2.5%). The UK farms surveyed in the present study used a wide range of breeding strategies with varying combinations of natural service and artificial insemination with conventional or sexed semen (Boulton *et al*., 2015b). These were associated with differing costs and fertility rates, leading to variations in the cost per heifer estimated at £32.21 for farms using only natural service up to £102.61/heifer for farms using sexed semen followed by natural service (Boulton *et al*., 2015b).

Gabler *et al*. (2000) and Heinrichs *et al*. (2013) both divided rearing up into four time periods: 3 days until weaning, weaning to 6 months, 6 months until breeding and breeding to calving. Using a cost analysis spreadsheet, Heinrichs *et al*. (2013) calculated the cost per head per day at $2.17, $1.39, $1.67 and $1.89, respectively, for these four periods, so weaning to breeding was the least and birth to weaning the most expensive. In the present study only three time periods were used, of which birth to weaning was again the most expensive at £3.14±0.85/day, reflecting high costs of feed and labour for pre-weaned calves (Boulton *et al*., 2015a). The UK average age at weaning reported here was 62 days compared with 57 and 58 in the two American studies (Gabler *et al*., 2000; Heinrichs *et al*., 2013). The costs for heifer rearing are not therefore a smooth function, with heavy initial costs pre-weaning followed by gradual costs from this point onwards. This early period is also when the animals are most vulnerable to disease and mortality (Brickell *et al*., 2009).

The costs incurred from weaning to conception and conception to calving were both similar at £1.65±0.40 and £1.64±0.47/day, respectively. Using conception rather than the time of first breeding as the cut-off point will affect the relative lengths of the last two periods, particularly if conception rates are poor. The average age at first breeding and age at conception reported here were 15.7 (range 12 to 23 months) and 16.7 (12 to 23.5 months) months, respectively, but the figures were highly variable between farms (Boulton *et al*., 2015b). Accurate conception rates were hard to obtain due to the widespread use of natural service, with bulls present on 71% of the 101 farms surveyed. The average AFC was 784 days compared with 734 and 776 days in the American studies (Gabler *et al*., 2000; Heinrichs *et al*., 2013). The higher weaning age and AFC would also affect the way that costs were apportioned to the different periods.

All the studies showed consistently that delaying the AFC increases the overall cost of rearing. For example, Mohd Nor *et al*. (2012) found that heifers calving at 30 months of age had a rearing cost ~€400 (£318) greater than heifers calving at 24 months. In the present study for heifers calving at 30 months the mean total rearing costs were on average £658 higher than for those calving at 24 months. The greater difference may in part be attributed to the costs of calving (building, bedding, disinfection), colostrum, feeding, calf housing, labour and utilities in the first 2 weeks of the heifer’s life, which were not included in the Dutch study. Kingshay (2013) presented total estimated costs of rearing from birth to calving for 24, 30 and 36 months of £1271, £1601 and £1847, respectively. The Department of Agriculture and Rural Development North Ireland (DARDNI) Farm Business Data (Keatley, 2016) calculated that the gross margin for autumn born heifers was £67 more per animal for those with a 24 compared with a 30-month AFC. Tozer and Heinrichs (2001) estimated that reducing the AFC by 1 month lowered the cost of a replacement programme by 4.3%. The length of this period is determined to a large extent by the farmers’ decisions on plane of nutrition and reproduction management. Tozer (2000) concluded that a higher plane of nutrition incurred higher daily feed costs, but these costs were recouped when heifers calved at a younger age through savings on labour, housing and overall feed costs. It is, however, necessary for animals to have achieved an adequate body size before calving or milk production potential in the first lactation is compromised (Bach and Ahedo, 2008).

The seasonal calving pattern in this study had a strong association with the total cost, with herds calving in spring (February through April) and autumn (August through October) having the lowest mean rearing costs. Output from a dynamic programming model developed by Mourits *et al*. (1999) found that the lowest discounted rearing costs in the Netherlands were for heifers born from September through February and highest in the period from May through July. In the United Kingdom, the seasonal effect is largely related to the amount of time which animals can spend grazing, as this decreases the input costs of feed, housing and labour. There was also a seasonal difference in feed costs which were most highly discounted in the summer.

The DARDNI farm business data suggested a gross margin per autumn born heifer in Northern Ireland of £339 compared with £357 for a spring born heifer, both based on an AFC of 24 months (Keatley, 2016). In contrast, the current study reports a higher mean gross margin for autumn compared with spring calving herds (£586 and £457, respectively). Gross margins were extremely variable as they are dependent upon the market value for animals: this is influenced by season, pedigree including lineage of the sire, breed and number of animals on sale on that market day. As much of this information was difficult to obtain with any certainty during data collection, mean values were used in the analyses. The cost of mortality is also variable depending on the actual mortality rates on the farm. The higher DARDNI gross margins only included a 2% mortality allowance, whereas the mean mortality rate for heifers from birth to calving in mainland UK was 15% (Brickell *et al*., 2009).

The mean number of lactations it took to repay the investment in the rearing period was 1.5 lactations, in agreement with Bach (2011). Although this is not an unreasonably long period of time, studies on the survival of heifers through to third lactation have shown culling rates in first and second lactations of 19% and 24% (Brickell and Wathes, 2011), with the largest proportion of these due to infertility. High culling rates increase the risk that the heifer will be disposed of before it has repaid the cost of rearing and so started making a profit for the farm. The average number of lactations in the UK herd is 3.5 to 3.8 (DairyCo, 2009; Hanks and Kossaibati, 2012), thus the farms that are of most concern are the 10% that are not breaking even until at least the third lactation. These must rely on other sources of income outwith the dairy if they are to survive economically. The length of the repayment period is not only influenced by the cost of rearing, but also by the farmgate price of milk and the production output. The smaller the difference between revenue from milk production and variable costs, the smaller the contribution to the repayment of rearing costs. The length of repayment is also influenced by the subsequent fertility of the milking herd. A useful measurement to assess is the lifetime yield per day of life, as this accounts for the non-productive periods before first calving and between lactations. This was shown to peak at 14.4 kg/day for cows calving at 24 months, reducing to 10.8 kg/day for calving at 36 months (Wathes *et al*., 2014).

In conclusion, based on the outcomes from this study, the cost of rearing dairy heifers in Great Britain is highly influenced by the AFC, the amount of time that heifers spend grazing, the calving pattern of the herd, the size of the milking herd and the breed. A ‘one figure fits all’ approach is therefore unrealistic and unhelpful to dairy farmers when they are comparing their own cost of rearing against what is considered to be an industry average. One quarter of farms paid back the investment in their youngstock in the first lactation and a further 68% in the second lactation. The length of the repayment period was dependent upon the total cost of rearing and the difference between revenue per day from milk and daily variable costs. Farmers require clear guidance on the long-term consequences of their choice of heifer management system. This requires that they understand the main factors which affect the costs in each system.
